# Supplementary material for: The effect of reproductive, hormonal, nutritional and lifestyle on breast cancer risk among black Tanzanian women: A case control study
Source: PLoS One. 2022 Feb 9;17(2):e0263374. doi: 10.1371/journal.pone.0263374 (PMC8827470; doi:10.1371/journal.pone.0263374)
Supplement: S1 File — (PDF) [file pone.0263374.s001.pdf]

**S1:English version of BC risk questionnaire**

## Interview parameters

1. Questionnaire number…………………..
2. Date of interview ………………………….
3. Name of interviewer…………………………………………………….
4. Name of ward……………………………
5. Patient file number(MR no)M/A……………………(for cases)
6. Histology result +ve -ve Histology type…………………………..

## Respondent's characteristics

1. In which date, month and year were you born? ………………..Age (years) …….
2. What is the highest level of education you attained?
   A. No formal education
   B. Primary education
   C. Secondary education (O-level)
   D. Advanced secondary education(A-level)/college(certificate)
   E. Diploma/Advanced diploma
   F. University (degree/masters/PHD etc)
3. Residence
   A. Town
   B. Village
4. What is your occupation?
   A. Agriculture / Farming
   B. Business / Trade
   C. Housewife
   D. Civil servant
   E. Other (Specify)…………………………………
5. What is your marital status?
   A. Single
   B. Married
   C. Cohabiting
   D. Widow
   E. Divorced/Separated

6. How old were you when you began to menstruate?.........
7. Was your menses regular at 18 years of age?
    A. Yes
    B. No
8. Do you still have menses?
    A. Yes
    B. No
9. If no, how did menopause occur?..................................
10. How old were you when menopause occurred?
11. Have you used following medicines during menopause?
    A. Yes
    B. No
12. How long did you use the following medicines during menopause?
    A. ……..Months
    B. …….years
13. Have you ever used following medicines?
    A. Contraceptives
        i. Yes
        ii. No
    B. Hormones for treatment of infertility
        i. Yes
        ii. No

## Pregnancy, parity and breastfeeding

14. Have you ever been pregnant?
    A. Yes
    B. No
15. If YES, how many times?......... (include live birth, still birth, miscarriages and abortions)
16. How old were you, when you were pregnant for the first time? ………..
17. Did you give birth?
    A. Yes
    B. No
18. How many times have you given birth?........ (include still births after 8th month)
19. How old were you when you delivered your first baby?......... (include still birth after 8th month)
20. Did you breast-feed?
    A. Yes (Go to question 21)
    B. No (Go to question 23)

21. If YES, how many children? …………………..
22. How long did you breast-feed, including mixed feeding?
    A. 1st baby …………………..
    B. 2nd baby ………………....
    C. 3rd baby …………………
23. History of breast lumps/ surgeries
    A. Yes
    B. No
24. History of obesity during adolescence
    A. Yes
    B. No
25. Someone in the family was diagnosed with breast cancer
    A. No (If unsure or could not remember)
    B. Yes (Mention the relationship with the patient)
        i. Father
        ii. Mother
        iii. Siblings
        iv. Grandmother / Grandfather
        v. Other relative …………………

## Active smoking

26. Do you smoke/ have you ever smoked cigarette in the past?
    A. Yes
    B. No (Go to question 29)
27. If yes, How old were you when you started to smoke? .........
28. How many cigarette do you smoke in a day? …….

## Passive smoking

29. Did / does someone smoke inside your living apartment?
    A. Yes
    B. No (Go to question 32)
30. How long does/did someone inside your apartment smoke...
    A. …….Months
    B. …….Years
31. How many hours per day do/did you spend in smoke filled apartment...
32. Does/ did someone smoke at your work place? ...
    A. Yes
    B. No

33. How long do you work at smoke filled work place?............

## Alcohol use

34. Do/did you drink alcoholic beverages?
    A. Yes ……
    B. No
35. How old were you when you started drinking habitually?.........
