# Supplementary material for: The effect of reproductive, hormonal, nutritional and lifestyle on breast cancer risk among black Tanzanian women: A case control study
Source: PLoS One. 2022 Feb 9;17(2):e0263374. doi: 10.1371/journal.pone.0263374 (PMC8827470; doi:10.1371/journal.pone.0263374)
Supplement: S2 File — (PDF) [file pone.0263374.s002.pdf]

Swahili Translation of the English questionnaire

I. Taarifa za mtafiti

1. Namba ya dodoso…………………..
2. Tarehe ya mahojiano ………………………………………………
3. Jina la mtafiti…………………………………………………
4. Jina la wodi……………………………………………………
5. Namba ya mgonjwa……………………………………………………
6. Majibu ya kipimo a)Kansa b) Hana kansa…(Aina ya kansa………………)

II. Taarifabinafsi za mshiriki

1. Ulizaliwa tarehe ngapi? Siku…. / mwezi…/Mwaka (Jaza umri kwa miaka)…….
2. Una kiwango gani cha elimu?
    A. Sikuwahi kwenda shule
    B. Elimu ya msingi
    C. Elimu ya sekondari (kidato cha nne)
    D. Elimu ya sekondari (kidato cha sita)/chuo ngazi ya cheti
    E. Chuo ngazi ya Stashahada
    F. Chuo kikuu (Shahada/uzamili/uzamivu)
3. Unaishiwapi?
    A. Jijini/ Mjini
    B. Kijijini
4. Unajishughulisha na nini?
    A. Kilimo
    B. Biashara
    C. Mama wa nyumbani
    D. Mwajiriwa
    E. Nyinginezo (Taja)…………………………………
5. Hali ya ndoa
    A. Sijaolewa
    B. Nimeolewa
    C. Ninaishi na mwanaume ila hatujafunga ndoa
    D. Mjane

E. Tumeachana/Tumetengana

6. Umeanza kupata hedhi ukiwa na miaka mingapi?..........….(miaka)
7. Bado unapata hedhi?
   - A. Ndiyo……….nenda swali la 14
   - B. Hapana
8. Kama hapana, ukomo wa hedhi ulitokeaje?..................
9. Ulikuwa na umri gani ukomo wa hedhi ulipotokea?.........................
10. Umewahi kutumia dawaza kuongeza vichocheo vya mwili vya kike kukinzana na ukomo wa hedhi?
    - A. Ndiyo
    - B. Hapana
11. Kama ndiyo umetumia kwa miaka mingapi?......................
12. Umewahi kutumia dawa zifuatazo?
    - A. Dawa za uzazi wa mpango
      - i. Ndiyo
      - ii. Hapana
    - B. Dawa za matibabu ya ugumba
      - i. Ndiyo
      - ii. Hapana
13. Umewahi kuwa mjamzito?
    - A. Ndiyo
    - B. Hapana ….nenda swali la 24
14. Kama ndiyo, mara ngapi?................(inahusisha mimba zilizoharibika)
15. Ulikuwa na umri gani ulipokuwa mjamzito kwa mara ya kwanza?............
16. Ulijifungua? ……………(inahusisha mimba zaidi ya miezi 8)
    - A. Ndiyo
    - B. Hapana
17. Umejifungua mara ngapi?.............(inahusisha mimba zilizoharibika baada ya miezi 8)
18. Ulikuwa na umri gani ulipojifungua kwa mara ya kwanza?.........
19. Je, ulinyonyesha?
    - A. Ndiyo
    - B. Hapana
20. Uli/umenyonyesha watoto wangapi?................
21. Umenyonyesha kwa muda gani? (inahusisha unyonyeshaji na chakula)
    - A. Mtoto wa kwanza ……..
    - B. Mtoto wa pili…………
    - C. Mtoto watatu………
22. Una historia ya uvimbe kwenye matiti?
    - A. Ndiyo

B. Hapana

23. Una historia ya kuwa mnene wakatiwa balehe yako?
    A. Ndiyo
    B. Hapana
24. Je umewahi/ unavuta sigara?
    A. Ndiyo
    B. Hapana …. nenda swali la 31
25. Kama ndiyo, ulikuwa na umri wa miaka mingapi ulipoanza kuvuta?...............(miaka)
26. Ulikuwa/unavuta sigara ngapi kwa siku?........
27. Umewahi kuishi na mtu anayevuta sigara ndani ya nyumba moja?
    A. Ndiyo ………..
    B. Hapana…..nenda swali la 34
28. Kama ndiyo, Kwa muda gani?
    A. Miezi………………..
    B. Miaka ………………
29. Ulikuwa / unakaa muda gani ndani ya nyumba yenye moshi?............(masaa/siku)
30. Kuna mtu anavuta sigara kazini kwako?
    A. Ndiyo
    B. Hapana………nenda swali la 36
31. Unafanya kazi katika mazingira ya moshi wa sigara kwa muda gani kwa siku?...miaka?....
32. Una historia ya kunywa pombe?
    A. Ndiyo
    B. Hapana nenda swali la 38
33. Ulinza kunywa pombe na miaka mingapi?...........
34. Kuna ndugu yako amewahi kupatikana na kansa ya ziwa?
    A. Hapana
    B. Ndio (Taja uhusiano wako na huyo ndugu)
        i. Baba
        ii. Mama
        iii. Bibi / Babu
        iv. Ndugu mwingine (taja)………………………
