# Supplementary material for: The effect of reproductive, hormonal, nutritional and lifestyle on breast cancer risk among black Tanzanian women: A case control study
Source: PLoS One. 2022 Feb 9;17(2):e0263374. doi: 10.1371/journal.pone.0263374 (PMC8827470; doi:10.1371/journal.pone.0263374)
Supplement: Attachment — Submitted filename: Rebutal letter.docx [file pone.0263374.s006.docx]

**MMUHIMBILI UNIVERSITY OF HEALTH AND ALLIED SCIENCES**

**SCHOOL OF MEDICINE**

***DEPARTMENT OF SURGERY***

P.O. Box 65001

DAR ES SALAAM

TANZANIA

Web: http://www.muhas.ac.tz

Telephone: +255-22-2150302/6 Ext: 1135

Direct Line: +255-22-2153241

Telefax: +255-22-2150465

E-mail: [hdsur@muhas.ac.tz](mailto:hdsur@muhas.ac.tz)

9^th^ October, 2021

Dear editor

Thanks again for giving us the opportunity to critically scrutinize our references and for your tireless effort to have this work published with your respected journal. Secondly, we are grateful for the reviewers for their good work to make the manuscript a possibility.

a) I have gone through the reference list one by one looking at whether they are available online and are retrievable, and secondly to establish if the citations were correct.
b) We now can confidently say that all our references meet the journal standards and are retrievable through Journal, year, issue, page and DOI or URL (for the GLOBOCAN data on Tanzania). The GLOBOCAN country sheets were the only retrievable source of accurate cancer statistics for Tanzania that we were able to identify in English literature.
c) We have made several alterations to the existing reference by introducing 7 new references
   i. #3 – The original reference was not clearly depicting the status of BC as being second to cervical cancer in SSA.
   ii. #5 – 7: To clearly depict the changing trend of cancer globally with a shift to low and middle income countries.
   iii. #14 – Correctly depicts the protective role of breast feeding on breast cancer.
   iv. #25 – 26: in the previous version, the citation was confusing as it was not addressing obesity with breast cancer. This new citation correctly relates Obesity to breast cancer risk.
d) The remaining references have either remained in the position with their number or remained in same position but with different number due to the introduction of the seven new citations.
e) Some changes have also been made in the entire document
   i. References are now in square brackets [ ] unlike the round brackets ( ).
   ii. Line 61 -62 has new content due to the new references inserted to enrich the document.
   iii. Line 283 – 286 and 296 – 298 has been re-written.

Yours sincerely

**Larry Akoko**

**Corresponding author**

Senior Lecturer

Department of surgery

Muhimbili University of Health and Allied sciences
