# Supplementary material for: The effect of reproductive, hormonal, nutritional and lifestyle on breast cancer risk among black Tanzanian women: A case control study
Source: PLoS One. 2022 Feb 9;17(2):e0263374. doi: 10.1371/journal.pone.0263374 (PMC8827470; doi:10.1371/journal.pone.0263374)
Supplement: Attachment — Submitted filename: Reviewer comments.docx [file pone.0263374.s004.docx]

Reviewer’s comments:

The submitted manuscript entitled (The effect of reproductive, hormonal, nutritional and lifestyle risk on breast cancer among black Tanzanian women: A case control Study) aimed to define some environmental factors that may play a role in the breast cancer such as hormones and nutrients according to the lifestyle.

The manuscript is a case study and well-presented and is a representative for the factors under investigation.

There are few minor comments:

1- Line 159: as mentioned that cases were significantly older and came from rural residences. However, the results showing that the number and percentage of urban is more than the rural in both control and cases.
2- For the age parameter, it is difficult to consider the average as a realistic so as I understood that it is analyzed later in details. However, it is still confusing about the age between 12 and 45.
3- The discussion part requires some modification to be more solid and informative.
4- The lifestyle part of discussion especially the part of residency as the results in table 2 showed that the more cases and controls came from urban in contrast to the discussion part.
